# Does the guided online cognitive behavioral therapy for insomnia “i-Sleep youth” improve sleep of adolescents and young adults with insomnia after childhood cancer? (MICADO-study): study protocol of a randomized controlled trial

**DOI:** 10.1186/s13063-021-05263-z

**Published:** 2021-04-26

**Authors:** Shosha H. M. Peersmann, Annemieke van Straten, Gertjan J. L. Kaspers, Adriana Thano, Esther van den Bergh, Martha A. Grootenhuis, Raphaële R. L. van Litsenburg

**Affiliations:** 1 Princess Máxima Center for Pediatric Oncology, Heidelberglaan 25, Utrecht, 3584 CS The Netherlands; 2 Emma Children’s Hospital, Amsterdam UMC, Vrije Universiteit, Pediatric Oncology, Cancer Center Amsterdam, Amsterdam, The Netherlands; 3 Department of Clinical, Neuro, and Developmental Psychology, Faculty of Behavioural and Movement Science & Amsterdam Public Health Research Institute, Vrije Universiteit Amsterdam, van der Boechorststraat 7, Amsterdam, 1081 BT The Netherlands

**Keywords:** Adolescent, Young adult, Childhood cancer, Insomnia, CBT, eHealth, Randomized controlled trial

## Abstract

**Background:**

Adolescents and young adults who had childhood cancer are at increased risk for insomnia, due to being critically ill during an important phase of their life for the development of good sleep habits. Insomnia is disabling and prevalent after childhood cancer (26–29%) and negatively impacts quality of life, fatigue, pain, and general functioning and is often associated with other (mental) health problems. Insomnia and a history of childhood cancer both increase the risk of adverse health outcomes, posing a double burden for adolescents who had childhood cancer. The first-line treatment for insomnia is cognitive behavioral therapy for insomnia (CBT-I). However, access to this type of care is often limited. The guided online CBT-I treatment “i-Sleep” has been developed to facilitate access via online care. i-Sleep is shown effective in adult (breast cancer) patients, but it is unknown if iCBT-I is effective in pediatric oncology.

**Methods/design:**

We developed a youth version of i-Sleep. Our aim is to evaluate its effectiveness in a national randomized-controlled clinical trial comparing iCBT-I to a waiting-list control condition at 3 and 6 months (*n* = 70). The intervention group will be also assessed at 12 months to see whether the post-test effects are maintained. Adolescents and young adults aged 12–30 years with insomnia, diagnosed with (childhood) cancer, currently at least 6 months since their last cancer treatment will be eligible. Outcomes include sleep efficiency (actigraphic), insomnia severity (self-report), sleep and circadian activity rhythm parameters, fatigue, health-related quality of life, perceived cognitive functioning, chronic distress, depressive and anxiety symptoms, and intervention acceptability.

**Discussion:**

Insomnia is prevalent in the pediatric oncology population posing a double health burden for adolescents and young adults who had childhood cancer. If guided iCBT-I is effective, guidelines for insomnia can be installed to treat insomnia and potentially improve quality of life and the health of adolescents and young adults who had childhood cancer.

**Trial registration:**

NL7220 (NTR7419; Netherlands Trial register). Registered on 2 August 2018

## Background

Sleep problems are common after childhood cancer with up to a third reporting significant sleeping disturbance [1, 2]. One particular type of sleep problem which is among the most prevalent ones is insomnia. Insomnia is characterized by difficulty in initiating or maintaining sleep for more than 3 nights a week for more than 3 months which significantly impacts daily life functioning [3]. Insomnia is associated with a lower health-related quality of life (HR-QoL), more fatigue, and worsened psychosocial and neurocognitive functioning [4–8]. In the long run, chronic insomnia is related to increased risk of adverse health outcomes, e.g., musculoskeletal pain, other mental disorders, and cardiovascular disease [9–12]. Childhood cancer survivors are already at increased risk of adverse health outcomes [13]. An additional insomnia diagnosis might therefore pose a double health burden. Furthermore, insomnia developed during childhood tends to become chronic, with up to 88% of adolescents with current insomnia, having a history of insomnia [14, 15]. Adolescents and young adults who had childhood cancer may even be at increased risk for insomnia, being critically ill during a phase of life that is important in the development of good sleep habits [8].

The increased risk of insomnia in adolescents, age-group defined as 12–25 years old [16], and young adults, age-group 19–30 years old [17, 18], who had childhood cancer might be explained within the conceptual model of insomnia: the three-factor (3P-) model. In this model, insomnia develops and is maintained through predisposing, precipitating, and perpetuating factors [19]. Predisposing factors in insomnia entail genetic vulnerability, but also major changes in the sleep architecture that occur during adolescence within circadian and homeostatic bioregulation processes [20]. Other hallmarks of adolescence are also risk factors for insomnia, such as higher stress reactivity and emotional problems due to hormonal changes in puberty, school stress, increased electronic media use, and heightened caffeine intake [21]. All provide a soil for insomnia, which can develop through additional precipitating factors.

Precipitating factors within the context of childhood cancer contain stressful life events, such as the cancer diagnosis and following treatment periods, but also medication and hospitalizations leading to sleep disruption. Being critically ill for a prolonged period of time can lead to change of sleep habits, with e.g. (re)occurrence of daytime napping, increased sleep fragmentation, and less physical activity, which eventually leads to a disruption of the circadian sleep-activity rhythm [8, 22–24]. In general, these sleep problems subside over time. However, not all patients recover. While during treatment up to 75% of patients report sleep problems, after treatment and into survivorship, symptoms remain in 28–29% [1, 2, 25]. In these cases, insomnia is maintained by perpetuating factors, such as the continued disruptive sleeping behaviors and increased hyperarousal. Hyperarousal is a heightened state of distress playing a role in maintaining insomnia and is one of the proposed underlying mechanisms [26]. The context of adolescents and young adults who had childhood cancer therefore entails several risk factors that increase the chance of development and maintenance of insomnia. However, currently, limited interventions for insomnia in adolescents in pediatric oncology are available.

The first-line treatment for insomnia in adults is cognitive behavioral therapy for insomnia (CBT-I), which is effective, shows less side effects, and sustains better long-term effects compared to pharmacological treatments [27, 28]. Compared to adults, the treatment of insomnia in adolescents has been less studied [21, 29]. So far, three randomized controlled trials showed the effectiveness of either face-to-face or online CBT-I for insomnia in adolescents [30, 31]. Furthermore, although there are hypotheses about potential treatment mechanisms of CBT-I for insomnia in adolescents, they are also yet to be validated. In childhood cancer, only one pilot study with a small sample of survivors aged (15–40 years) studied face-to-face and videoconference CBT-I showing adequate feasibility and effectivity [32]. However, even if evidence-based interventions exist, accessibility of those interventions might be low. This is the case for face-to-face CBT-I for insomnia due to a shortage in trained therapists [28]. New developments of online therapy might provide an answer to this problem, having the advantage of flexibility and overcoming geographical boundaries [33]. Online CBT-I (iCBT-I) shows similar effect sizes in treating insomnia in adults compared to face-to-face CBT-I [34]. Additional guidance by an online sleep coach remains important to increase adherence and motivation [35].

The treatment investigated in this study is based on i-Sleep, a guided 5-week iCBT-I program including common CBT-I treatment principles [36, 37]. i-Sleep was shown effective in improving insomnia, and secondary outcomes such as depressive symptoms and HR-QoL, in general population adults in a clinical trial and in an open-cohort study with adult breast cancer patients [38–40]. However, it is unknown if iCBT-I is also effective in adolescents and young adults with insomnia after childhood cancer. We therefore adapted the guided iCBT-I program to fit the adolescent age group and will investigate if “i-Sleep youth” can effectively treat insomnia in adolescents who had childhood cancer.

### Aims and hypotheses

The objective of this trial is to evaluate the effectiveness of the online guided cognitive-behavioral therapy “i-Sleep youth” in treating insomnia in adolescents and young adults who had childhood cancer compared to a waiting-list-control condition in a 1:1 parallel-group superiority RCT. Our hypothesis is that the intervention will improve sleep efficiency (actigraphy) and insomnia severity (self-report) on the short-term compared to the control group, which is maintained on the long-term up to a year after the intervention. Furthermore, we expect that patients in the intervention group will also show improvement on other actigraphy-based sleep parameters (sleep onset latency, wake after sleep onset, total sleep time). We will also assess two non-parametric circadian rhythm parameters, in which we expect that the intervention group will show increased stability and less fragmentation of the 24-h sleep-activity rhythm. Other outcomes we hypothesize to be improved after the intervention are HR-QoL, fatigue, (perceived) cognitive functioning, and depressive and anxiety symptoms compared to the control group. We also aim to study the acceptability of the online intervention in the adolescent target group. At last, we aim to explore the treatment mechanism in this population by studying the following mediators: chronic hyperarousal (defined as chronic distress) and disrupted daytime rhythm (napping). These mediating treatment factors were found in CBT-I for adult insomnia populations [41] and might also be mediating factors in treating insomnia after childhood cancer (higher chronic distress and disrupted rhythm due to being ill).

## Methods/design

### Study design

**Fig. 1:**
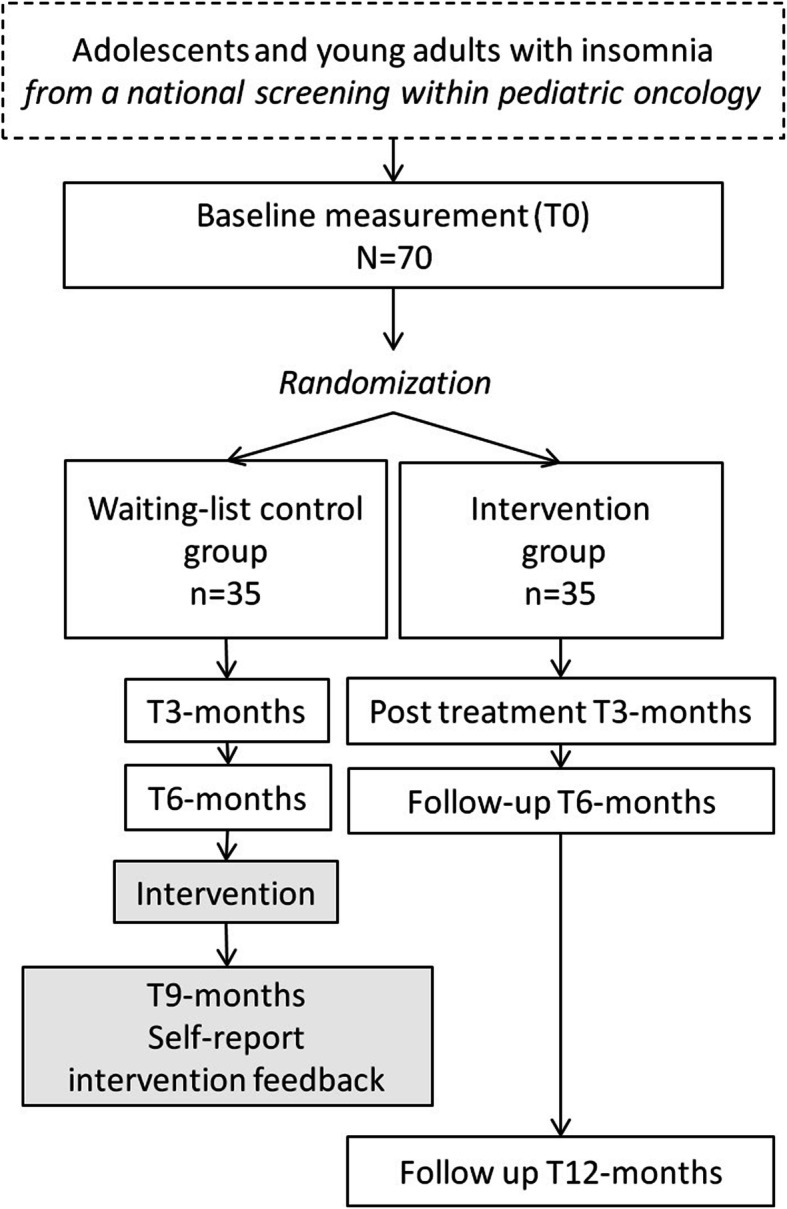
Design of the trial and study flow

The MICADO-study (Managing Insomnia after Childhood cancer in ADOlescents) is a national randomized controlled clinical trial. The trial will be coordinated from the Princess Máxima Center for pediatric oncology, which is the single national pediatric cancer center in the Netherlands. Patients with insomnia during pediatric cancer follow-up or survivor care will be randomized to either immediate access to the online intervention or the waiting-list control group. Participants in the waiting-list control group will receive the online treatment after 6 months. The design of the trial and study flow is shown in Fig. 1. This trial has been approved by the Medical Ethics Committee of UMC Utrecht (NL65009.041.18) and registered at the Netherlands National Trial Register (Trial NL7220 (NTR7419)). Patient inclusion started in January 2019.

### Participants: inclusion and exclusion criteria

Patients are eligible when they fulfill the following criteria: have insomnia symptoms of at least subthreshold clinical severity (Insomnia Severity Index-score ≥ 8; ISI, see the “Outcomes” section), aged between 12 and 30 years old, diagnosed with (childhood) cancer or a low-grade brain tumor and at least 6 months since their last cancer treatment (since insomnia during treatment can be directly related to therapy and recover spontaneously). Exclusion criteria are (a) receiving palliative therapy; (b) not sufficiently fluent in Dutch; (c) significant cognitive impairment; (d) comorbidities that affect sleep or can be exacerbated by the sleep restriction in CBT-I, e.g., severely diminished eye-sight (sleep-wake rhythm can be affected due to poor sight), acute schizophrenia or substance abuse, history of a seizure disorder diagnosis or having experienced a seizure within the past 12 months; (e) pregnancy or patients who just had a newborn < 6 months, since sleep problems often occur in pregnancy and parents of a newborn; (f) shift work employment; (g) suicidality; (h) current psychological treatment for psychopathology (e.g., substance abuse, psychosis) or sleeping disorders; and (i) severe sleep disorders other than insomnia and untreated sleep-breathing disorders (SBD). Patients that use sleep medication such as benzodiazepines and melatonin will not be excluded since they may still benefit from the intervention. The use of sleep medication will, however, be monitored throughout the study, as a secondary outcome of the intervention.

### Recruitment and randomization

Participants in the MICADO-study are recruited through a national insomnia screening in childhood cancer patients and survivors at the Princess Máxima Center and the affiliated shared-care hospitals. Patients with (subthreshold) insomnia (Insomnia Severity Index-score ≥ 8; ISI) that are interested in participating in the trial are sent an information package and are approached by the research team for a telephonic screening including additional information and an eligibility check. After the telephonic screening participants are sent an informed consent form and will be approached 2 weeks later to provide opportunity for questions. When participants return the form with written consent, it is signed off by RvL or SP and a copy will be sent back. After written consent (in patients < 16 years consent given by patients as well as parents/legal caregivers) is given, patients are sent the baseline measurement package. Participants will be randomized to the intervention or waiting list group with a 1:1 ratio, using minimization techniques, and will be stratified on the use of sleep medication. Randomization will take place after the baseline assessment and is outsourced to an independent party using the randomization program ALEA. Due to the nature of the study, blinding of the participants or the researchers is not possible.

### Description of the guided online CBT for insomnia intervention: i-Sleep youth

**Fig. 2:**
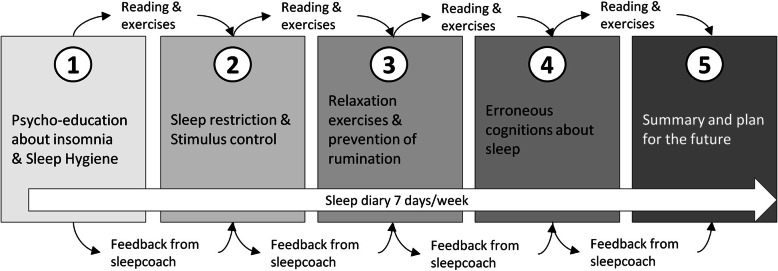
The topics of the five lessons and intervention flow

I-Sleep is an online therapy to treat insomnia based on common CBT-I principles with non-synchronous online guidance by a sleep coach. The Internet intervention is developed at the Vrije Universiteit (author AvS) based on CBT-I protocols including psycho-education, stimulus control, sleep restriction, relaxation, and cognitive restructuring. These elements are offered in 5 sessions in 5–8 weeks through written texts, videos, patients vignettes, and exercises. A sleep diary app is complimentary used to guide the monitoring the sleep behavior and sleep restriction. For more information about the development of the original intervention, see Van Straten et al. [40]. The topics of the five lessons and intervention flow are shown in Fig. 2. The intervention is weekly guided by an online coach with a psychosocial background supervised by a sleep expert. We adapted the original version of i-Sleep into “i-Sleep youth” to accommodate the target group adolescents (12–30 years) after childhood cancer. The instruction text, videos, logo, images, and four patient vignettes (two video and two written vignettes per session) were adjusted to adolescent language use and examples entailing both younger adolescent stories including going to school, as older adolescents who just started studying or working. Age-appropriate themes were added: attention for screen use, gaming, drugs, and energy drinks [21]. As parents play a key role in the life of mainly the younger adolescents, an intervention factsheet for parents with a summary and support tips was developed. Within the Dutch law of Agreement on Medical Treatment Act, adolescents who are 16 years or older are considered competent to enter a medical agreement independently. Therefore, within i-Sleep youth, the factsheet is optional for adolescents above 16 years of age. Parents of 13–15 years olds will automatically receive the factsheet. After the adaptation phase, i-Sleep youth was evaluated on target-group suitability and usability by three independent child-sleep experts, the Dutch childhood cancer parent-patient organization, and eight adolescents in a suitability and usability evaluation. The evaluation results were used for the final adjustments of i-Sleep youth.

#### Intervention group: i-Sleep youth condition

Patients in the intervention group will start i-Sleep directly after randomization for 5–8 weeks. They will receive an e-mail with a username, password, and a welcome message from the sleep coach. During the treatment, the patient will work on homework and exercises. When the homework is finished, the coach will receive a notification. Within a week, the coach will provide online feedback on the homework. The coaching and feedback are aimed at commenting on exercises, motivating the participants to persist with the exercises necessary for the behavioral changes and to clarify the information in the module. In the current trial, SP will coach the patients, supervised by AS, experienced in insomnia treatment, and a licensed CBT child-psychologist within the Princess Maxima Center.

#### Control group: waiting list condition

Patients in the control group will be randomized to a waiting list of 6 months after which they are offered the intervention. They are asked not to seek treatment for sleeping problems (either behavioral or pharmacological) during the waiting period. Adherence to this request will be assessed at follow-up time points. After 6 months they will be offered the intervention on which they will provide feedback via an intervention feedback questionnaire and contribute to studying intervention acceptability. The effectiveness of i-Sleep will not be assessed in this group.

### Assessments

The outcomes will be evaluated at baseline (T0), 3 months (T3), and 6 months (T6) after randomization. Patients in the intervention will additionally be assessed at 12 months (T12). See Table 1 for an overview of assessments. Acceptability of i-Sleep will be measured following the intervention. All self-report questionnaires will be offered through a secure website, www.hetklikt.nu. This secure internet environment is in use in patient care as well as in pediatric (oncology) care in the Netherlands [42, 43]. Paper questionnaires will be available upon request to the participants. The baseline choice of modality (paper-pencil or online) will be retained for all time points.

**Table 1:** Study outcome measurements and time points

Measurement	Instrument	Nr. of items	Time-points			
			Baseline (T0-months)	Post treatment (T3 months)	Follow-up T6 months	Follow-up T12* months
**Primary outcomes**						
Sleep efficiency (SE)	Actigraph and sleep log	–	X	X	X	X
Insomnia Severity	ISI	7	Assessed in screening	X	X	X
**Secondary outcomes:**						
Sleep parameters: TST, TTB, SOL, WASO, napping	Actigraph and sleep log		X	X	X	X
Circadian rhythm parameters: Fragmentation (IV) and intradaily stability (IS)	Actigraph and sleep log		X	X	X	X
Health-Related (HR) quality of life	PedsQL - teens 13–18 years - young adult 19–30 years	23	X	X	X	X
Fatigue	CIS	20	X	X	X	X
Depressive and anxiety symptoms	HADS	14	X	X	X	X
Perceived cognitive functioning	Peds-PCF-SF	10	X	X	X	X
Chronic stress	CSQ-CA	19	X		X	X
General questionnaire	Pain Sleep medication use	2	X	X	X	X
Intervention feedback	Satisfaction questionnaire, adherence	20		X*/X**		

Notes.*only intervention-condition, **at 9 months for control-condition. *TST* total sleep time, *TTB* total time in bed, *SOL* sleep onset latency, *WASO* wake after sleep onset, *ISI* Insomnia Severity Index, *CIS* Checklist Individual Strength, *HADS* Hospital Anxiety and Depression Scale, *Peds-PCFSF* Peds Perceived Cognitive functioning Short Form, *CSQ-CA* Chronic Stress Questionnaire for Childs and Adolescents

### Outcomes

#### Primary outcomes

##### Sleep efficiency (SE)—based on actigraphy

Sleep efficiency (SE) based on a 7-day wrist actigraphy measurement is a primary outcome of this trial. SE can be calculated by dividing total sleep time (TST) by the total time the person spent in bed (× 100%). SE contains information on difficulties falling asleep as well as difficulties staying asleep and is therefore often considered the best primary outcome measure as it represents different types of sleep problems [44]. Average SE throughout the week will be calculated using information gathered from the actigraph and accompanied sleep log [45]. For more information on actigraphy methods, see paragraph “sleep parameters.”

##### Insomnia severity—self-report questionnaire

*Insomnia severity* is additionally a primary outcome and will be evaluated using the Insomnia Severity Index (ISI) [46]. The ISI is a 7-item self-report questionnaire that assesses the nature, severity, and impact of insomnia. The dimensions are severity of sleep onset, sleep maintenance and early morning waking problems, sleep dissatisfaction, interference of sleep difficulties with daytime functioning, noticeability of sleep problems by others, and distress caused by sleep difficulties. It is rated on a 5-point Likert scale (total score 0–28). A score of 0–7 indicates absence of insomnia, 8–14 sub-threshold insomnia, 15–21 moderate insomnia, and 22–28 severe insomnia. Internal consistency is excellent in community as well as clinical samples (Cronbach’s alpha 0.90–0.91). Acceptable reliability is shown in cancer survivors [47]. A decrease of 8 points is considered a relevant clinical change in insomnia severity [46].

#### Secondary outcomes

##### Sleep parameters based on actigraphy and sleep log

Sleep parameters from actigraphy and sleep log of our interest are total sleep time (TST), total time in bed (TIB), sleep onset latency (SOL), wake after sleep onset (WASO), napping, number of awakenings. Actigraphy measures sleep-wake rhythm from the presence or absence of wrist movement, by a nonintrusive wristwatch style device that patients will wear 24 h a day for 7 days [48]. This method yields typical sleep-pattern measures. This method has been widely used; reliability and validity have been established in children and adults [49–51]. A paper-pencil self-report sleep log containing a few items on sleep-wake schedule will be kept additionally measuring: in-bed and out-bed times, naps, and time not wearing actigraph (e.g., during showering). The actigraph that will be used in this study is the GTX3+, a commercially available valid, and reliable device for detecting sleep/wake diurnal patterns [52]. Accompanying standardized software ActiLife will be used to extract sleep outcomes with the Sadeh algorithm, developed for use in children as well as in young adults [53].

##### Circadian rhythm parameters—based on actigraphy

To obtain circadian rhythm parameters, a non-parametric analysis is performed on raw wrist-actigraphy data [54, 55]. Two parameters are calculated including (1) *Stability of circadian rhythm* indicated by intradaily stability (IS), which represents the similarity of day-night patterns over time by the strength of synchronization with environmental 24-h cues named zeitgebers. Stability of rhythm measured with IS ranges from 0 to 1, in which 1 is perfect synchronization with zeitgebers.

(2) *Fragmentation of circadian rhythm* is indicated by intradaily variability (IV), which is the amount of shifting between rest and activity. Ideally, a 24-h rhythm entails low fragmentation with a period of a daily activity as opposed to a period of rest at night. Fragmentation measured with IV ranges from 0 to 2, with a higher IV indicating a more fragmented rhythm.

##### Self-report questionnaires

*HR quality of life (HR-QoL)* will be assessed using the acute version of the Pediatric Quality of Life Inventory (PedsQL) Generic core scales (23 items). The multidimensional scales are physical, emotional, social, and school/work functioning. The 5-point Likert scale investigates to what extent the problem occurred in the last week. Each answer is reversed scored and rescaled to 0–100 scale (0 = low, 100 = high HR-QoL). The questionnaire demonstrates adequate reliability and validity in adolescents and Dutch references are available [56, 57].

*Fatigue* will be measured with the Checklist Individual Strength (CIS) subscale “fatigue severity” (8 items), scored on a 7-point Likert scale (range 8–56; higher score indicates more fatigue). CIS-F has shown adequate reliability and discriminative validity [58, 59] and has previously been used as a treatment outcome in online CBT studies in adolescents for fatigue [60, 61].

*Depressive and anxiety symptoms* will be assessed with the Hospital Anxiety and Depression Scale (HADS), measuring depressive (7 items, score range 0–21) and anxiety (7 items, score range 0–21) symptoms. Items are rated from 0 (no distress) to 3 (maximum). It has shown adequate psychometric properties in Dutch adult populations and in adolescents [62, 63].

*Perceived Cognitive Functioning* is assessed with the short form of the Peds-PCF. The Peds-PCF measures perceived neurocognitive functioning. The Dutch short form of the Peds-PCF has recently been tested through IRT modeling and showed satisfactory psychometric properties. Scores range from 10 to 50, in which a higher score indicates better neurocognitive functioning [64].

*Chronic distress* in the past 3 months will be measured using the Chronic Stress Questionnaire for Children and Adolescents (CSQ-CA). This 17-item self-report questionnaire has been validated in adolescents and has adequate psychometric properties [65]. It consists of 4-point Likert scales, 1 = not true for me at all to 4 = completely true for me (score range 17–68) in which a higher score indicates more distress in the past 3 months.

An *additional questionnaire* assesses subjective pain (on a 10-point visual analog scale (VAS)) and sleep medication use at T3, T6, and T12. Use of sleep medication, either prescribed or over the counter, is measured with a sleep medication 4-Likert scale item: “During the past month, how often have you taken medicine (prescribed or “over the counter”) to help you sleep?” (0 = not in the past month, 4 = three or more times in the past month). Furthermore, sociodemographic and clinical variables are derived from the national screening prior to the MICADO-study.

*Post-treatment intervention feedback* and *satisfaction with i-Sleep* will be evaluated after completing the intervention with a similar acceptability procedure as in the previous i-Sleep trial in breast cancer patients by Dozeman and colleges [38] existing of a 20-item satisfaction questionnaire developed by our research group. In addition, data will be extracted from the e-health platform to evaluate adherence, and acceptability by evaluating intervention feedback per session. These two questions are included at the end of each session: “How do you evaluate the session?” (1–10) and “Did this session help you” (1–10).

### Sample size calculation

The sample size calculation for the RCT is based on the comparison of sleep efficiency (SE) between the *iCBT-I* group and waiting list group after 3 months (T3) using the program PASS version 16. Previous studies have shown an improvement in SE varying between 4.3 and 11%, with a standard deviation of about 11.5%. In adults that received the i-Sleep intervention, baseline SE was around 67% [40]. Mean iCBT-I improvements in SE vary between 5 and 11% [31, 40]. Standard deviations were similar pre- and post-intervention, about 11.5% [40]. In this study, sample sizes of 29 in each group will achieve 80% power with a significance level of 0.05, a slope of *λ*^1^ = 8.0, with a standard deviation of *σ*^*y*^ = 11.5, and a standard deviation of the condition of *σ*^*x*^ = 0.50 using linear regression sample size calculation. Adjustment for the baseline score was not possible due to limited literature [66]. Previous studies show an average drop-out after treatment of 20% [39, 40, 67, 68]. Taking into account this potential drop-out rate of 20%, a total sample of 70 (35 per group) is necessary to achieve adequate power for the per-protocol effectiveness analysis.

### Statistical analyses

Intention-to-treat as well as per-protocol analysis will be performed. A linear regression model adjusted for baseline score will be estimated to answer our primary study aim: the effectiveness of iCBT-I on SE and insomnia severity (ISI-score) in the iCBT-I group and the WL posttreatment (T3). The differences in SE and ISI at T3 between both groups will also be expressed in Cohen’s d effect sizes. For our secondary study aims, the clinical relevant response from the intervention effect posttreatment will be investigated by estimating a logistic regression model using yes/no clinical improvement based on the self-reported insomnia severity, since this represents both sleep problems, as daytime consequences of insomnia. A clinically relevant improvement is defined based on the self-report ISI as a decrease of ≤ 8 points between T0 and T3 [46, 67]. Due to the presence of repeated measurements a mixed linear model will be estimated to investigate the long-term effects. Mediation analyses at T3 are performed with the regression-based PROCESS macro [69] in which the potential mediational effect of chronic stress and napping on treatment effectivity on SE and ISI at T3 is tested. Missing data will be imputed under the appropriate missing data analysis assumptions, e.g. leading to multiple missing data imputation [70]. All analyses will be performed using SPSS and R.

### Composition of the coordinating center and trial steering committee

The research team entails childhood oncologists and fellows, psychologists, epidemiologists, and research assistants. Weekly meetings are held with the core research team providing day-to-day support including RvL, SP, and research assistants. Monthly the trial is supervised by MG, GK, AvS, and the Trial and Data Center (TDC) of the Princess Maxima Center. Stakeholders are involved through yearly cooperating with the patient-parent organization VOKK.

### Monitoring

This trial is monitored by the monitoring company Julius Clinical based on a low-risk study profile. The monitor consists of an initiation visit, two monitor visits (after 10 and after 50 inclusions), and a close-out visit (Julius Clinical reference: 18-534). No interim analyses are performed due to the low-risk profile. Furthermore, each year the accredited medical ethical committee will be updated on the progress of the trial.

## Discussion

Insomnia is common in adolescents and young adults who had childhood cancer and poses a high burden that can persist for a long time if left untreated. The cancer diagnosis and treatment in itself may increase the risk of developing insomnia, while ongoing dysfunctional sleeping habits and distress may perpetuate insomnia in the long run [8, 23, 24, 71–73]. In adult cancer survivors the efficacy of internet cognitive behavioral therapy for insomnia (iCBT-I) shows promising results [74]. iCBT-I might provide an answer to the paucity in access to insomnia care and shows similar effect sizes compared to face-to-face CBT-I in adults [34]. iCBT-I might be a potential treatment for insomnia after childhood cancer. Therefore, this paper presents the design of the MICADO-study, a randomized controlled trail, to investigate the efficacy of the iCBT-I intervention “i-Sleep youth” in adolescents and young adults who had childhood cancer.

This trial has several strengths and limitations. Firstly, it contributes to the expanding literature of the effectivity of iCBT-I in adolescents with insomnia and is the first RCT studying its effectiveness for adolescents with insomnia after childhood cancer. Furthermore, the trial has a high external validity, since we include a nationwide patient population derived from a national screening in all pediatric oncology centers in the Netherlands. However, this has the disadvantage that there might be a self-selection bias in participants responding to the national screening invitation, e.g., specific subgroups, such as with a lower education level, socioeconomic status, or language barrier are less likely to respond [75, 76]. Another strength of this study is that we include both subjective and objective measures of sleep, which is recommended in insomnia treatment trials [44]. A limitation is that the coach is also the investigator, not allowing for blinding or masking, which potentially poses an expectancy bias. However, the coaching contact only entails guidance online in the self-help program or via post mail, so investigator contact to elicit this bias is limited. At last, the waiting-list control is a non-active control group which might inflate treatment effects [77]. Furthermore, the waiting-list control receives treatment after 6 months, which limits comparison of long-term effects of iCBT-I only up to 6 months. However, currently, there is no standard care-as-usual for this patient group and a non-active waiting list is commonly used to study cognitive behavioral interventions [78].

Overall, there is a need for interventions for adolescents with insomnia after childhood cancer. Additional insomnia on top of being a childhood cancer survivor may put an already vulnerable group for adverse outcomes, even more at risk for impaired physical and psychosocial health. Currently, the availability of first-line treatment cognitive behavioral therapy for insomnia is limited. An online guided modality might provide an answer. Therefore, the online cognitive behavior therapy “i-Sleep youth” for adolescents and young adults with insomnia after childhood cancer was adapted and will be studied in this trial. Insomnia treatment has the potential to contribute to enhancing health-related quality of life and psychosocial functioning in this adolescent pediatric oncology population.

## Trial status

At the time of submission of this paper (protocol version number 4, 11-12-2020), subject recruitment is still ongoing. Inclusion started in 24 January 2019 and study completion is expected in September 2021.

## Data Availability

The data collection is ongoing. When the dataset for analyses is completed, the datasets used and/or analyzed during the current study are available from the corresponding author on reasonable request.

## Abbreviations

CBT-I: Cognitive behavioral therapy for insomnia
CSQ-CA: Chronic Stress Questionnaire for Children and Adolescents
CIS: Checklist Individual Strength
DSM-IV: Diagnostic and Statistical Manual of Mental Disorders
DSPS: Delayed-sleep phase disorder
HADS: Hospital Anxiety and Depression Scale
HR-QoL: Health-related quality of life
HSDQ: Holland Sleep Disorder Questionnaire
iCBT-i internet-based: Cognitive behavioral therapy for insomnia
ISI: Insomnia Severity Index-score
IS: Intradaily stability
IV: Intradaily variability
MICADO: Managing Insomnia after Childhood Cancer in Adolescents
PedsQL: Pediatric Quality of Life Inventory
RCT: Randomized controlled trial
SBD: Sleep breathing disorders
SE: Sleep efficiency
TST: Total sleep time
TIB: Total time in bed
SOL: Sleep onset latency
VAS: Visual analog scale
WASO: Wake after sleep onset
